# Targeted Nanoparticles: an Innovative Modality in the Treatment of Cancer

**DOI:** 10.64898/2025.12.01.691687

**Published:** 2025-12-02

**Authors:** Hyung-Gyoo Kang, Bryon Upton, Ryan W. Holly, Racheal Upton, Sheetal Mitra, Jean-Hugues Parmentier, Ann F. Mohrbacher, Yong-Mi Kim, Timothy J. Triche, Jon O. Nagy

## Abstract

Despite progress made in the development of anticancer therapeutics, traditional small-molecule chemotherapeutics often struggle to overcome toxicity, efficacy, and off-target effects. These specific issues can be overcome by either encapsulating the drug or by targeting it directly to the tumor cell. Here, we describe a novel targeted nanoparticle (referred to as a nano-antibody-drug conjugate Targeted Nanosphere or nADC/TNS), based cancer therapeutic platform that can improve the efficacy of a broad range of existing therapeutics. Targeting is antibody-directed, as with antibody-drug conjugates (ADCs). Still, the payload per antibody is vastly greater by orders of magnitude (a thousand for nADC/TNS versus two to eight for ADCs). The nADC/TNS consists of an approximately 80 nm drug-filled nanoparticle composed of phospholipids, cholesterol, and UV cross-linkable diacetylene lipids. We describe the preparation, characterization, and evaluation of nADC/TNS as a novel, versatile, and effective treatment modality for cancer and potentially other diseases. This report focuses on data with nADC/TNS variants NV101 (anti-CD99 targeted, doxorubicin-filled), NV102 (anti-CD19 targeted, doxorubicin-filled), and NV103 (anti-CD99 targeted, irinotecan-filled). We investigated NV101 and NV103 in a mouse model with implanted and metastatic Ewing tumors (ES). NV101 demonstrated significant tumor burden reduction while NV103 induced complete ablation of ES tumors. NV102 demonstrated complete ablation of chemotherapy-resistant relapsed adult lymphocytic leukemia (ALL). These results document the potential superior efficacy of antibody-targeted nanoparticles containing a variety of small-molecule payloads, compared to their free molecule equivalents.

## Full Text Availability

The license terms selected by the author(s) for this preprint version do not permit archiving in PMC. The full text is available from the preprint server.

